# Comparison of McGrath Videolaryngoscope versus Macintosh Laryngoscope in Tracheal Intubation: An Updated Systematic Review

**DOI:** 10.3390/jcm12196168

**Published:** 2023-09-24

**Authors:** Pasquale Sansone, Luca Gregorio Giaccari, Antonio Bonomo, Francesca Gargano, Caterina Aurilio, Francesco Coppolino, Maria Beatrice Passavanti, Vincenzo Pota, Maria Caterina Pace

**Affiliations:** Department of Woman, Child and General and Specialized Surgery, University of Campania “Luigi Vanvitelli”, 81100 Naples, Italy; lucagregorio.giaccari@gmail.com (L.G.G.); bonus93@libero.it (A.B.); f.gargano@unicampus.it (F.G.); caterina.aurilio@unicampania.it (C.A.); francesco.coppolino@unicampana.it (F.C.); mariabeatrice.passavanti@unicampania.it (M.B.P.); vincenzo.pota@inwind.it (V.P.); mariacaterina.pace@unicampania.it (M.C.P.)

**Keywords:** McGrath videolaryngoscope, Macintosh laryngoscope, tracheal intubation, airway management

## Abstract

(1) Background: In the last few years, many randomized controlled trials (RCTs) have compared direct Macintosh laryngoscopy with McGrath videolaryngoscopy in order to assess the potential benefits of the latter; the results were sometimes controversial. (2) Methods: We conducted a comprehensive literature search to identify our articles according to inclusion and exclusion criteria: to be included, each study had to be a prospective randomized trial or comparison between the McGrath videolaryngoscope and the Macintosh laryngoscope in an adult population. We did not include manikin trials or studies involving double-lumen tubes. (3) Results: 10 studies met the inclusion criteria necessary. In total, 655 patients were intubated with the McGrath and 629 with the Macintosh. In total, 1268 of 1284 patients were successfully intubated, showing equivalent results for the two devices: 648 of 655 patients with the McGrath videolaryngoscope and 620 of 629 patients with the Macintosh laryngoscope. No differences were noted in terms of hemodynamic changes or the incidence of adverse events. (4) Conclusions: We can assert that the McGrath videolaryngoscope and Macintosh laryngoscope, even if with equivalent tracheal intubation results, supplement each other.

## 1. Introduction

The McGRATH™ MAC videolaryngoscope is an essential device with a high-resolution video camera placed within an angulated single-use blade of adjustable length [1,2]; it is designed to give a better laryngeal view than that achieved via direct laryngoscopy with a Macintosh laryngoscope. The McGrath videolaryngoscope has the potential to be useful in difficult laryngoscopy situations, even if compared with a conventional Macintosh laryngoscope; however, its efficacy in tracheal intubation was found to be inconsistent [3].

In recent years, many randomized controlled trials (RCTs) and meta-analyses have compared direct Macintosh laryngoscopy with videolaryngoscopy. There have been several RCTs comparing tracheal intubation success rates between the McGrath and Macintosh laryngoscopes. Some studies suggested that the McGrath has higher success rates compared to the Macintosh laryngoscope [3], while other studies showed lower success rates [4]. This may be attributable to the device itself or to the degree of experience in the use of indirect laryngoscopes compared to the normal direct approach of the Macintosh laryngoscope. With regard to tracheal intubation, the time taken to use the McGrath in a normal airway is shorter than that of the Macintosh laryngoscope [5]. On the other hand, more time was required to use the McGrath in intubation inpatients with immobilized cervical spine or obstetric patients [1,6,7]. Furthermore, another study showed that the McGrath provides a better view of the glottis compared to the Macintosh laryngoscope [8,9]. Other studies state that there is no superiority of one laryngoscope over the other [10,11].

Aims. Although there are several studies comparing Macintosh direct laryngoscopy to videolaryngoscopy for tracheal intubation in adults, it is unclear whether the McGrath has any advantages. In our work, we performed a systematic review of several RCTs to compare the effectiveness between the McGrath videolaryngoscope and the Macintosh laryngoscope for tracheal intubation in an adult population. We evaluated different aspects of intubation (rate of successful tracheal intubation, duration of the intubation maneuvers, number of attempts, need for external maneuvers or other alternative techniques, hemodynamic changes and incidence of adverse events) with the McGrath versus Macintosh laryngoscope in order to assess the real benefits of videolaringoscopy.

## 2. Materials and Methods

Protocol and registration. This systematic review was prepared following the recommendations of the Preferred Reporting Items for Systematic Reviews and Meta-Analyses (PRISMA) statement [12]. Randomized controlled trials that compared the effectiveness of the McGrath videolaryngoscope and the Macintosh laryngoscope for tracheal intubation in an adult population were included. This review is not registered in the international prospective register of systematic reviews (PROSPERO). This article reviews previously conducted studies and does not contain human or animal studies performed by either of the authors.

Eligibility criteria. The search was performed following the Population, Intervention, Comparison and Outcome (PICO) criteria (see Table 1). Patients older than 18 years undergoing general anesthesia were considered as the population (P); the intervention (I) was tracheal intubation using the McGRATH™ MAC videolaryngoscope; the comparison (C) concept was standard tracheal intubation using the Macintosh laryngoscope; and the rate of successful tracheal intubation, the duration of the intubation maneuvers, the number of attempts, the need for external maneuvers or other alternative techniques, the hemodynamic changes and the incidence of adverse events (AEs) were considered the outcomes (O) for this systematic review. We included randomized controlled trials published from September 2017 to February 2023. In a previous systematic review, Hoshijima et al. performed a comprehensive literature search until August 2017 [13]. We did not include manikin trials or studies involving double-lumen tubes.

Literature search. The search was conducted across the main electronic databases (Medline, EMBASE, PubMed, Google Scholar and Cochrane Library—CENTRAL). Other relevant studies were selected from the reference lists. We used a combination of terms such as “McGrath videolaryngoscope”, “Macintosh laryngoscope”, “direct laryngoscope”, “tracheal intubation”, “successful tracheal intubation” and “randomized controlled trials”. No restrictions on language or type of publication were considered. The most recent search was performed in March 2023.

Primary outcomes. The primary outcome was the rate of successful tracheal intubation between the McGRATH™ MAC videolaryngoscope and the Macintosh laryngoscope.

Secondary outcomes. The duration of intubation maneuvers, the number of attempts, the need for external maneuvers or other alternative techniques, the hemodynamic changes and the incidence of adverse events were the secondary outcomes.

Quality of the study. Each study was evaluated using the COSMIN checklist. The risks of bias of the study are due to incomplete outcome data or selective outcome reporting and other potential threats to validity.

Data synthesis and analysis. Data were abstracted using a uniform data collection form by one of the authors. The RCTs found were reviewed by all authors and any questions were discussed amongst all authors on regular video-conference meetings.

The data extracted included the following:

1. Number of participants involved in the study;
2. Age, sex and body mass index (BMI) of participants;
3. Type of operation;
4. ASA classifications;
5. Airway status, using Modified Mallampati score: class I—visualization of soft palate, uvula, fauces and pillars; class II—visualization of soft palate, major part of uvula and fauces; class III—visualization of soft palate and base of uvula; and class IV—visualization of hard palate.
6. Cormack and Lehane classification of glottic view: grade I—visualization of entire vocal cords; grade II—visualization of posterior part of the laryngeal aditus; grade III—visualization of epiglottis only; and grade IV—no glottic structures seen.
7. Duration of intubation, defined as the time between placement of the endotracheal tube (ETT) between the dental arches and the appearance of the first capnographic curve.
8. Number of attempts. Each attempt corresponded to the time between the introduction of the laryngoscope into the oral cavity and its removal. Intubation failure was defined as the inability to intubate after three attempts. In the case of failure, an alternative technique was used at the discretion of the anesthesiologist.
9. Number and type of optimization maneuvers, like the use of a bougie, cricoid pressure and a second assistant, were recorded.
10. Hemodynamic changes, considering the change in heart rate (HR) and blood pressure (BP) before and after intubation.
11. Adverse events (AEs), such as oxygen desaturation, dental damage, oro-pharyngeal trauma, esophageal intubation, postoperative hoarseness and postoperative sore throat.

Statistical analysis. In addition to descriptive statistics, an analysis was performed using the Statistical Package for Social Science (SPSS release 24.0, 2016; IBM, Chicago, IL, USA). The Shapiro–Wilk test was used to demonstrate the normal distribution. Success rate equivalence between the two devices was calculated as the difference between the success rates and its two-sided 99% CI. If the CI for the difference between the success rates was within the equivalence range of ±5%, the two devices were deemed equally successful. Mann–Whitney’s U test, the chi-squared test and Fisher’s exact test were used to detect significant differences between groups during the analysis of study population characteristics and secondary endpoints, as appropriate. The association between the success rate and potentially influencing factors (gender, body mass index, age, cervical spine immobilization and indication for airway management) was assessed using multiple logistic regression analysis. A *p*-value of 0.01 was deemed statistically significant throughout the study. Correspondingly, the CIs were 99%.

## 3. Results

**Figure 1:**
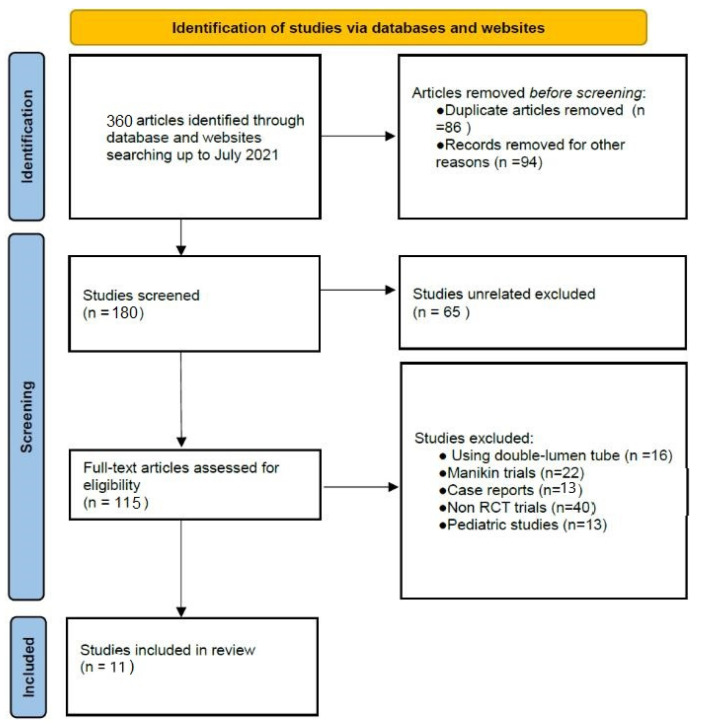
Flow diagram of study selection process.

Our search strategy identified 360 articles; of these, 180 studies were excluded because of duplicate articles (*n* = 86) and other reasons of incompatibility (*n* = 92). At a first check of 180 publications identified, 65 studies were excluded because they were unrelated. A total of 115 potentially eligible publications remained, of which 105 were excluded because 16 used a double-lumen tube, 22 were manikin trials, 13 were case reports, 40 were non-RCT trials and 13 were pediatric studies. Overall, 11 studies met all the inclusion criteria and were included in this systematic review. The results from the literature search and the study selection process are shown in Figure 1.

The characteristics of the studies included in this review are reported in Table 2.

Two authors (P.S. and L.G.G.) independently evaluated the quality of the RCTs. None of the 11 studies had a high risk of bias.

In the included studies, 1379 patients underwent tracheal intubation: 700 (51%) patients were intubated with the McGrath videolaryngoscope and 679 (49%) with the Macintosh laryngoscope. All patients underwent elective surgery requiring orotracheal intubation; only two articles included patients with potentially difficult airways [17,21]. Difficult airways were as defined those of obese patients or in emergency scenarios. In all the trials, the intubation was performed by a trained anesthetist who had experience with the use of the Macintosh laryngoscope and the McGrath videolaryngoscope.

The mean age was 47.3 ± 13.4 years in the DL group and 48.4 ± 14.1 years in the VL group. Sex was reported for all studies, except for that of Thion et al. [16]: there were 651 males (323 in DL group versus 328 in VL group) and 611 females (304 in DL group versus 307 in VL group). Regarding the BMI, it was 30.3 ± 10.4 kg/m^2^ in the DL group and 30.4 ± 10.0 kg/m^2^ in the VL group. No statistically significant differences between groups were reported in terms of demographic characteristics.

The Mallampati scores were reported in half of the studies and they were similar in both groups. In the DL group, patients had Mallampati scores of class I (*n* = 124), class II (*n* = 79), class III (*n* = 59) and class IV (*n* = 6). In the VL group, patients had Mallampati scores of class I (*n* = 122), class II (*n* = 87), class III (*n* = 52) and class IV (*n* = 3).

Patient characteristics and Mallampati scores are summarized in Table 3.

Other characteristics such as thyro-mental distance, maximum mouth opening, mobility of the cervical spine and state of the upper incisors were reported randomly and for this reason not considered in the evaluation of the airways.

**Figure 2:**
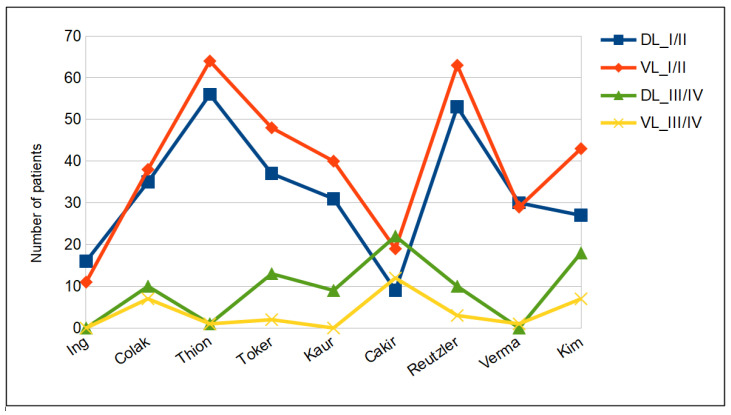
Cormack–Lehane grade.

**Figure 3:**
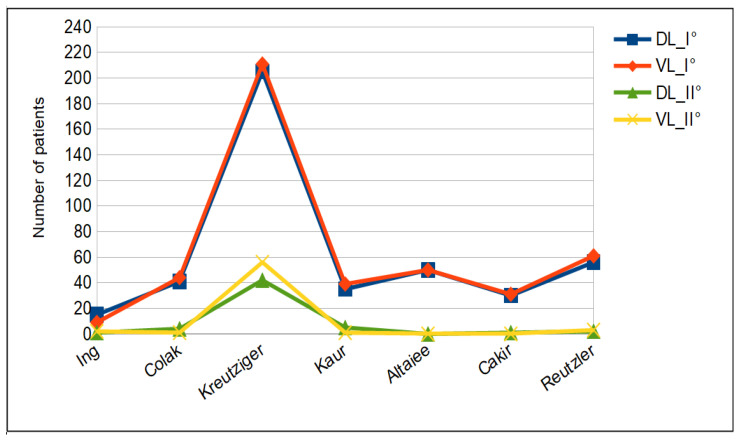
Number of attempts for patients.

**Figure 4:**
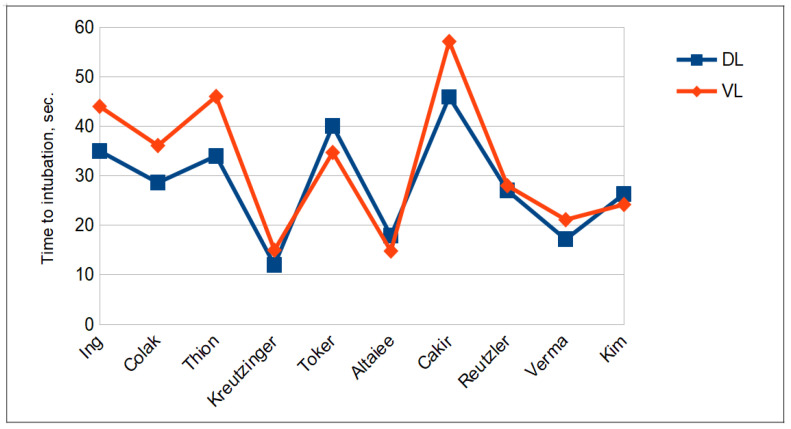
Time to intubation, seconds.

Tracheal intubation. The difference in Cormack–Lehane grading was not significant between the DL and VL groups (see Figure 2). Cormack–Lehane grades I and II were observed in 294 of the patients (43.3%) in the DL group and 355 of those (50.7%) in the VL group (*p*-value = 0.2054). As shown in Figure 3, intubation was successful on the first attempt in 489 (72.0%) patients in the McGrath MAC group and 479 (68.4%) patients in the Macintosh group. The duration of intubation was longer in the VL group (29.8 ± 14.5 s) than in the DL group (28.4 ± 10.2 s) but the difference did not reach statistical significance (see Figure 4). On the contrary, BURP was performed in 39 (6.2%) patients in the DL group and in 23 patients (3.4%) in the VL group. Alternative techniques were used 27 times versus 12 times in the DL and VL groups, respectively.

Overall, 98.5% of patients were successfully intubated, showing equivalent results for the two devices: 692 of 700 patients (98.9%; CI, −0.6% to 2.6%) with the McGrath videolaryngoscope and 667 of 679 patients (98.2%; CI, −0.6% to 2.6%) with the Macintosh laryngoscope. Thus, the difference in the success rates was 0.7%, and the 99% CIs for the difference in the success rates (99% CI, −2.58 to 3.39) were within the supposed equivalence range of ± 5%. The remaining 21 patients were successfully ventilated with alternative airways: 10 (47.6%) with a larynx tube, 4 (19.0%) with a laryngeal mask, 4 (19.0%) with a coniotomy and 2 not reported (14.4%).

Hemodynamic changes. The baseline heart rate (HR) in the DL group was 81.2 ± 13.8 bpm and in the VL group it was 79.5 ± 12.1 bpm with a *p*-value > 0.05. There was a slight increase in heart rate in the DL group at 5 min, which was not statistically significant. The VL group showed a reduced heart rate variability.

The mean arterial pressure (MAP) in the DL group was 95.9 ± 10.3 mmHg before induction and 89.0 ± 1.6 mm Hg after intubation with a *p*-value = 0.145. The VL group showed no change in MAP from baseline and there was no statistical significance. The VL group showed a decrease in MAP compared to the DL group, but this was not statistically significant (*p*-value = 0.346).

The systolic arterial pressure (SAP) in the DL group was 126.2 ± 20.7 mmHg before induction and 111.9 ± 11.9 mmHg after intubation (*p*-value = 0.223). In the VL group, SAP showed values of 125.2 ± 21.0 mmHg and 108.5 ± 10.4 mmHg and there was no statistical significance (*p*-value = 0.185).

**Figure 5:**
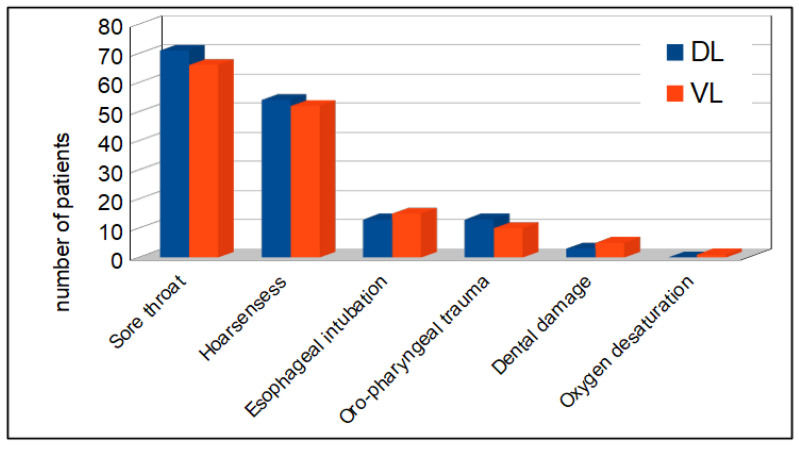
Adverse events in DL and VL groups.

Adverse events. Postoperative hoarseness occurred, respectively, in 8.6% and 7.9% of the patients in the DL and VL groups; sore throat occurred in 11.3% using the Macintosh laryngoscope and 10.1% using the McGrath videolaryngoscope. The difference did not reach statistical significance whatever the group. Other complications were less frequent. Esophageal intubation accounted for less than 3% in both groups. Dental damage and oral-pharyngeal trauma were equally distributed between the DL and VL groups, affecting 18 and 16 patients, respectively. Only one case of oxygen desaturation was reported using the McGrath videolaryngoscope. Adverse events are shown in Figure 5.

## 4. Discussion

Nowadays, the increasing need to face difficulties in airway management has resulted in a higher use of videolaryngoscopy; however, even if it seems to be superior to direct laryngoscopy for tracheal intubation, its use remains controversial [13].

We worked to obtain an updated point of view about the use of the McGrath videolaryngoscope versus the Macintosh laryngoscope, examining the latest articles published. Considering the rate of successful tracheal intubation between the two devices, no statistical difference was found in terms of successful tracheal intubation between the McGrath videolaryngoscope and the Macintosh laryngoscope. We did not observe any difference in terms of number of intubation attempts or failed intubations. According to the Cormack–Lehane classification, glottic view is similar between the two groups. If it had been different, significant differences in favor of the videolaryngoscope would probably have been observed in routine clinical practice. The Cormack–Lehane classification was validated as a predictor of difficult tracheal intubation for direct laryngoscopy using a Macintosh laryngoscope. The McGrath videolaryngoscope, in most cases, offers a good view of the glottis; the difficulties in tracheal intubation do not derive from the visualization of the glottis, but from the manipulations of the tube. The duration of intubation was longer when using the McGrath videolaryngoscope but the difference did not reach statistical significance. In the studies included, all providers were more experienced in intubating with the Macintosh laryngoscope but not with the McGrath videolaryngoscope, and this may explain the longer intubation time using the videolaryngoscope. In Hoshijima et al., the McGrath videolaryngoscope required a longer intubation time and this suggests that the intubation time is significantly prolonged with the McGrath, possibly due to clinicians’ experience in using laryngoscopes [13]. As demonstrated by various studies, the ability to perform mask ventilation and intubation and the time taken for intubation improve significantly with increasing experience [24,25]. Therefore, experience plays a major role in reducing intubation attempts, minimizing the risk of complications and reducing the use of additional intubation devices.

Some studies report that while videolaryngoscopy improves vocal cord visualization, it prolongs the time required for intubation and increases the number of intubation attempts [26,27,28].

Another aim of our study was to evaluate the hemodynamic response to endotracheal intubation by using the conventional Macintosh laryngoscope and the McGrath videolaryngoscope. The increase in the HR observed after intubation in the DL group can be considered mild and clinically insignificant. The SAP and MAP also showed a decrease from the baseline in both the groups. The anesthesia regime followed for intubation was probably accountable for this. We believe that there is no difference between the two laryngoscopes in terms of hemodynamic parameters.

In this review, we could not detect any difference between the Macintosh laryngoscope and the McGrath videolaryngoscope in terms of adverse events. No severe life-threatening complications were recorded. This is in contrast to previous studies where the percentage of patients with severe life-threatening complications was higher using videolaryngoscopy, while there was no significant difference between the groups for mild to moderate life-threatening complications [29]. This may be due to the similar median duration of the orotracheal intubation procedure with the two techniques.

Limitations. The main limitation of our study is that the anesthesiologists performing the laryngoscopy and intubation could not be blinded to the devices used in the studies. Furthermore, there is a learning curve for videolaryngoscope as all anesthesiologists are mainly trained to use the Macintosh laryngoscope. The experience of providers was not addressed or controlled in the included studies. Second, some studies included in our review have small sample sizes. Third, Cormack–Lehane is a validated instrument for glottic exposure assessment in direct laryngoscopy but not in videolaryngoscopy. Finally, the chosen studies differ in their definitions of rate of successful intubation, duration of maneuvers, number of attempts, hemodynamic changes and even adverse events.

## 5. Conclusions

In conclusion, from the data extracted from our study, even if equivalent tracheal intubation results were found for the two devices, we can assert that the two devices supplement each other. Further studies are needed to underline and confirm the benefits of the McGrath VL in daily anesthetic practice.

## Figures and Tables

**Table 1:** PICO criteria.

**Population**	Patients of at least 18 years undergoing general anesthesia.
**Intervention**	Tracheal intubation using the McGRATH™ MAC videolaryngoscope.
**Comparator**	Tracheal intubation using the Macintosh laryngoscope.
**Outcomes**	Rate of successful tracheal intubation, the duration of the intubation maneuvers, the number of attempts, the need for external maneuvers or other alternative techniques, the hemodynamic changes and the incidence of adverse events.
**Study type**	Randomized controlled trials (RCTs).
**Time**	From September 2017 to February 2023.

**Table 2:** Studies’ characteristics.

	Year	N° of Patients (DL/VL)	Type of Surgery
Ing et al. [14]	2017	27 (16/11)	Elective surgery
Colak et al. [15]	2018	90 (45/45)	Elective surgery
Thion et al. [16]	2018	122 (57/65)	Elective surgery
Kreutziger et al. [17]	2019	514 (247/267)	Emergency
Toker et al. [18]	2019	100 (50/50)	Elective cesarean section
Kaur et al. [19]	2020	80 (40/40)	Elective surgery
Altaiee et al. [20]	2020	100 (50/50)	Elective surgery
Çakir et al. [21]	2020	62 (31/31)	Elective bariatric surgery
Ruetzler et al. [22]	2020	129 (63/66)	Elective surgery
Verma et al. [23]	2020	60 (30/30)	Elective cardiac surgery
Kim et al. [24]	2023	95 (50/45)	Elective surgery

**Table 3:** Patient characteristics and Mallampati scores.

KERRYPNX	DL	VL
Male/Female	323/304	328/307
Age (years)	47.3 ± 13.4	48.4 ± 14.1
BMI (kg/m^2^)	30.3 ± 10.4	30.4 ± 10.0
Mallampati		
– I	124	122
– II	79	87
– III	59	52
– IV	6	3

## Data Availability

Datasets derived from public resources and are available on request. Contact person: Pasquale Sansone (e-mail: pasquale.sansone@unicampania.it).

## References

[1] Taylor A.M., Peck M., Launcelott S., Hung O.R., Law J.A., MacQuarrie K., McKeen D., George R.B., Ngan J. (2013). The McGrath^®^ series 5 videolaryngoscope vs Macintosh laryngoscope: A randomised, controlled trial in patients with a simulated difficult airway. Anesthesia.

[2] Ng I., Sim X.L., Williams D., Segal R. (2011). A randomised controlled trial comparing the McGrath^®^ videolaryngoscope with the straight blade laryngoscope when used in adult patients with potential difficult airways. Anesthesia.

[3] Sargin M., Uluer M.S. (2016). Comparison of McGrath^®^ serie 5 video laryngoscope with Macintosh laryngoscope: A prospective, randomised trial in patients with normal airways. Pak. J. Med. Sci..

[4] Frohlich S., Borovickova L., Foley E., O’Sullivan E. (2011). A comparison of tracheal intubation using the McGrath or the Macintosh laryngoscopes in routine airway management. Eur. J. Anaesthesiol..

[5] Kwak H.J., Lee S.Y., Cho S.H., Kim H.S., Kim J.Y. (2016). McGrath video laryngoscopy facilitates routine nasotracheal intubation in patients undergoing oral and maxillofacial surgery: A comparison with Macintosh laryngoscopy. J. Oral. Maxillofac. Surg..

[6] Ilyas S., Symons J., Bradley W.P.L., Segal R., Taylor H., Lee K., Balkin M., Bain C., Ng I. (2014). A prospective randomised controlled trial comparing tracheal intubation plus manual in-line stabilisation of the cervicale spine using the Macintosh laryngoscope vs the McGrath^®^ series 5 videolaryngoscope. Anaesthesia.

[7] Arici S., Karaman S., Doğru S., Karaman T., Tapar H., Özsoy A.Z., Kaya Z., Süren M. (2014). The McGrath series 5 videolaryngoscope versus the Macintosh laryngoscope: A randomised trial in obstetric patient. Turj. J. Med. Sci..

[8] Liu Z.J., Guo W.J., Ma C., Huang Y.G. (2016). Comparison of McGrath series 3 and Macintosh laryngoscopes for tracheal intubation in patients with normal airway by inexeperienced anesthetists: A randomized study. Medicine.

[9] Wallace C.D., Foulds L.T., McLeod G.A., Younger R.A., McGuire B.E. (2015). A comparison of the ease of tracheal intubation using a McGrath MAC^®^ laryngoscope and a standard Macintosh laryngoscope. Anaesthesia.

[10] Walker L., Brampton W., Halai M., Hoy C., Lee E., Scott I., McLernon D. (2009). Randomized controlled trial of intubation with the McGrath series 5 videolaryngoscope by inexperienced anaesthetists. Br. J. Anaesth..

[11] Foulds L.T., McGuire B.E., Shippey B.J. (2016). A randomised cross-over trial comparing the McGrath^®^ series 5 videolaryngoscope with the Macintosh laryngoscope in patients with cervical spine immobilisation. Anaesthesia.

[12] Moher D., Liberati A., Tetzlaff J., Altman D.G. (2009). The PRISMA Group. Preferred Reporting Items for Systematic Reviews andMeta-Analyses: The PRISMA Statement. BMJ.

[13] Hoshijima H., Mihara T., Maruyama K., Denawa Y., Takahashi M., Shiga T., Nagasaka H. (2018). McGrath videolaryngoscope versus Macintosh laryngoscope for tracheal intubation: A systematic review and meta-analysis with trial sequential analysis. J. Clin. Anesth..

[14] Ing R., Liu N., Chazot T., Fessler J., Dreyfus J.F., Fischler M., Le Guen M. (2017). Nociceptive stimulation during Macintosh direct laryngoscopy compared with McGrath Mac videolaryngoscopy: A randomized trial using indirect evaluation using an automated administration of propofol and remifentanil. Medicine.

[15] Colak F., Ozgul U., Erdogan M.A., Kayhan G.E., Erdil F.A., Colak C., Durmus M. (2019). Comparison of hemodinamic responses and QTc intervals to tracheal intubation with the McGRATH MAC videolaryngoscope and Macintosh direct laryngoscope in elderly patients. Kaohsiung J. Med. Sci..

[16] Thion L.A., Belze O., Fischler M., Le Guen M. (2018). Comparison of the ease of tracheal intubation using a McGrath Mac videolaryngoscope and a standard Macintosh laryngoscope in normal airways: A randomised trial. Eur. J. Anaesthesiol..

[17] Kreutziger J., Hornung S., Urschl W., Doppler R., GVoelckel W., Trimmel H. (2019). Comparing the McGrath Mac Video Laryngoscope and Direct Laryngoscopy for Prehospital Emergency Intubation in Air Rescue Patients: A Multicenter, Randomized, Controlled Trial. Crit. Care Med..

[18] Toker M.K., Altıparmak B., Karabay A.G. (2019). Comparison of the McGrath video laryngoscope and macintosh direct laryngoscope in obstetric patients: A randomized controlled trial. Pak. J. Med. Sci..

[19] Kaur G., Gupta S., Metha N., Dhingra J.S. (2020). Comparative Evaluation of McGrath MAC, Truview Video Laryngoscopes and Macintosh Laryngoscope for Endotracheal Intubation in Patients Undergoing Surgery under General Anaesthesia. Anesth. Essays Res..

[20] Altaiee A.H., Hassen H.A., Fadeel S.J. (2020). Video laryngoscopy versus direct laryngoscopy on time of orotracheal intubation in normal adult in elective surgeries. Med. Sci..

[21] Çakir M., Özyurt E. (2020). Comparison of direct laryngoscope and McGrath videolaryngoscope in terms of glottic view and hemodynamics in bariatric surgery. Turk. J. Med. Sci..

[22] Ruetzler K., Rivas E., Cohen B., Mosteller L., Martin A., Keebler A., Maheshwari K., Steckner K., Wang M., Praveen C. (2020). McGrath Video Laryngoscope Versus Macintosh Direct Laryngoscopy for Intubation of Morbidly Obese Patients: A Randomized Trial. Anesth. Analg..

[23] Verma I., Verma C., Dhaked S., Sharma R. (2020). A Randomised Comparative Interventional Prospective Study of Intubation by Macintosh Laryngoscope Versus McGrath Video Laryngoscope in Patients Undergoing Cardiac Surgery. Anesth. Crit. Care.

[24] Kim J.Y., Park S., Oh M., Choi J.B., John H.J., Lee S.K., Choi Y.H. (2023). Comparison of the McGRATH^TM^ Video Laryngoscope and Macintosh Laryngoscope for Orotracheal Intubation in a Simulated Difficult Airway Scenario: An Open-Label, Randomized Clinical Trial. Medicina.

[25] Singhal S.K., Kaur K., Yadav P. (2021). A study to evaluate the role of experience in acquisition of the skill of orotracheal intubation in adults. J. Anaesthesiol. Clin. Pharmacol..

[26] Aziz M.F., Abrons R.O., Cattano D., Bayman E.O., Swanson D.E., Hagberg C.A., Todd M.M., Brambrink A.M. (2016). First-attempt intubation success of video laryngoscopy in patients with anticipated difficult direct laryngoscopy: A multicenter randomized controlled trial comparing the C-MAC D-blade versus the GlideScope in a mixed provider and diverse patient population. Anesth. Analg..

[27] Sulser S., Ubmann D., Schlaepfer M., Brueesch M., Goliasch G., Seifert B., Spahn D.R., Ruetzler K. (2016). C-MAC videolaryngoscope compared with direct laryngoscopy for rapid sequence intubation in an emergency department: A randomised clinical trial. Eur. J. Anaesthesiol..

[28] Ruetzler K., Imach S., Weiss M., Haas T., Schmidt A.R. (2015). Comparison of five video laryngoscopes and conventional direct laryngoscopy: Investigations on simple and simulated difficult airways on the intubation trainer. Anaesthesist.

[29] Lascarrou J.B., Boisrame-Helms J., Bailly A., Le Thuaut A., Kamel T., Mercier E., Ricard J.D., Lemiale V., Colin G., Mira J.P. (2017). Video Laryngoscopy vs Direct Laryngoscopy on Successful First-Pass Orotracheal Intubation Among ICU Patients: A Randomized Clinical Trial. JAMA.

